# A 17 gene panel for non‐small‐cell lung cancer prognosis identified through integrative epigenomic‐transcriptomic analyses of hypoxia‐induced epithelial–mesenchymal transition

**DOI:** 10.1002/1878-0261.12491

**Published:** 2019-05-29

**Authors:** Yue‐Lei Chen, Yihe Zhang, Junwen Wang, Na Chen, Weiying Fang, Jianing Zhong, Yi Liu, Rui Qin, Xinxin Yu, Zhongsheng Sun, Fei Gao

**Affiliations:** ^1^ Stem Cell Bank/Stem Cell Core Facility Institute of Biochemistry and Cell Biology Shanghai Institutes for Biological Sciences Chinese Academy of Sciences Shanghai China; ^2^ Agricultural Genomics Institute at Shenzhen Chinese Academy of Agricultural Sciences Shenzhen China; ^3^ E‐GENE Co., Ltd. Shenzhen China; ^4^ Institute of Genomic Medicine Wenzhou Medical University China; ^5^ Beijing Institutes of Life Sciences Chinese Academy of Sciences Beijing China

**Keywords:** biomarker, EMT, epigenetics, metastasis, non‐small‐cell lung cancer

## Abstract

As a critical feature of the tumor microenvironment, hypoxia is known to be a potent inducer of tumor metastasis, and it has been proposed that the initial steps in metastasis involve epithelial–mesenchymal transition (EMT). The strong correlation among hypoxia, EMT, and metastasis suggests that integrative assessment of gene expression and the DNA modification program of hypoxia‐induced EMT via high‐throughput sequencing technologies may increase our understanding of the molecular basis of tumor invasion and metastasis. Here, we present the genomewide transcriptional and epigenetic profiles of non‐small‐cell lung cancer (NSCLC) cells under normoxic and hypoxic conditions. We demonstrate that hypoxia induces EMT along with dynamic alterations of transcriptional expression and epigenetic modifications in both A549 and HCC827 cells. After training using a dataset from patients with invasive and noninvasive lung adenocarcinomas with an artificial neural network algorithm, a characteristic 17‐gene panel was identified, consisting of genes involved in EMT, hypoxia response, glycometabolism, and epigenetic modifications. This 17‐gene signature clearly stratified NSCLC patients with significant differences in overall survival across three independent datasets. Our study may be suitable as a basis for further selection of gene signatures to potentially guide prognostic stratification in patients with NSCLC.

**Abbreviations**
5hmC: 5‐hydroxymethylcytosine
5mC: methylcytosine
bHLH: basic helix‐loop‐helix
DEG: differentially expressed gene
DhMR: differentially hydroxyl‐methylated region
DMR: differentially methylated region
DNMT: DNA methyltransferase
EMT: epithelial–mesenchymal transition
FDR: false discovery rate
GO: gene ontology
HIF: hypoxia‐inducible factor
hMeDIP: hydroxymethylated DNA immunoprecipitation
HRE: hypoxia response element
KEGG: Kyoto Encyclopedia of Genes and Genomes
MBD: methyl binding protein domain
MeDIP: methylated DNA immunoprecipitation
NSCLC: non‐small‐cell lung cancer
RPKM: reads per kb per million reads
TET: ten‐eleven translocation
TSS: transcriptional start site

## 1 Introduction

Lung cancer is the leading cause of cancer death in China (Chen *et al*., 2016) and worldwide (Torre *et al*., 2016). Non‐small‐cell lung cancer (NSCLC) accounts for approximately 85% of all lung cancers, which are often diagnosed at an advanced stage and are associated with a poor prognosis. In contrast to the steady increase in survival for most cancers, the 5‐year relative survival for lung cancer remains unsatisfactory, mainly due to progressive metastasis and resistance to anticancer therapy (Torre *et al*., 2016). Therefore, an increased understanding of the molecular mechanisms underlying tumor invasion and metastasis is crucial for improving the early detection and treatment of this disease.

It has been proposed that the initial steps in the invasion–metastasis cascade involve an epithelial–mesenchymal transition (EMT) process, converting nonmotile epithelial tumor cells at a primary site into cells with migratory mesenchymal characteristics that are prone to invade other tissues at a distant site (Thiery *et al*., 2009). EMT can be induced either by a number of soluble cytokines or by varied physicochemical conditions in the tumor microenvironment, such as hypoxia (Le *et al*., 2006; Mittal *et al*., 2016). Hypoxia is a pathobiological hallmark of rapidly growing solid tumors, including NSCLC (Gilkes *et al*., 2014). When tumors outgrow the oxygen supply delivered by the vascular system, the low oxygen levels initiate the hypoxic response machinery in the cell. This response is mediated by a structurally conserved family of basic helix‐loop‐helix (bHLH) transcription factors known as hypoxia‐inducible factors (HIFs) (Mimeault and Batra, 2013). Most studies ascribe the hypoxic response to the binding of canonical HIFs to functional hypoxia response elements (HREs) within the promoter of molecular targets that potently drive EMT and tumor metastasis (Rankin and Giaccia, 2008). In addition to the stabilization and activation of these transcriptional regulators, epigenetics plays a crucial role in the cellular response of solid tumors to a hypoxic microenvironment (Hancock *et al*., 2015). Importantly, a series of epigenetic enzymes, including the ten‐eleven translocation (TET) DNA hydroxymethylases and histone demethylases, are regulated in an oxygen‐sensitive manner (Iyer *et al*., 2009; Tahiliani *et al*., 2009). Such epigenetic regulation may work jointly with HIF family members or even contribute in a more substantial way to maintain a variety of hypoxia‐mediated cellular functions that promote tumor progression and metastasis. Therefore, a detailed understanding of the molecular mechanism underlying hypoxia‐mediated responses in tumor cells has important implications for producing novel therapeutic strategies and potential biomarkers in the clinic.

Previous attempts to discover biomarkers in lung cancer have referred to molecular abnormalities, including genomic instability, DNA mutations, transcriptional noise and epigenetic aberrations (Vargas and Harris, 2016). However, despite the plethora of studies addressing potential single biomarkers for use in the diagnosis, prognosis, and epidemiology of lung cancer, very few of these markers have sufficed to enable clinical implementation. Over the past decade, the emergence of high‐throughput sequencing technologies has offered the promise of a comprehensive understanding of carcinogenesis. Given a strong correlation among hypoxia, EMT, and metastasis, integrative epigenomic–transcriptomic analyses of hypoxia‐induced EMT provide a possibility for identifying novel biomarkers in diagnosis, prognosis, and treatment of lung cancer. In the present study, we revealed the characteristic molecular signatures of hypoxic NSCLC cells by integrating epigenomic, transcriptomic, and HIF target analyses. A 17‐gene panel was identified as potential biomarkers for prediction of metastasis and prognosis in patients with NSCLC (workflow of data generation and analysis in Fig. S1).

## 2 Materials and methods

### 2.1 Cell culture and hypoxic exposure

The human NSCLC cell lines A549 (K‐RAS G12S), HCC827 (exon 19 deletion of EGFR), NCI‐H838 (K‐RAS amplified), NCI‐H1437 (wild‐type K‐RAS), and NCI‐H1975 (EGFR T790M) were obtained from the ATCC and cultured in medium supplemented with 10% fetal bovine serum (FBS) at 37 °C under 5% CO_2_ and 95% air. For the hypoxia experiments, the cells were first seeded at 8 × 10^3^ to 1 × 10^4^ cells/cm^2^ in normoxic conditions and then incubated under strictly controlled hypoxic conditions (1% O_2_) in a hypoxic chamber (ASTEC, Tokyo, Japan) for the desired time periods. Each experiment was repeated in triplicate.

### 2.2 Western blot analysis

Cell extracts were prepared in RIPA buffer and subjected to western blot analysis as described previously (Zhang *et al*., 2013). The primary antibodies described in this paper include antibodies against β‐actin (Sigma, St. Louis, MO, USA), ANGPTL4 (Chemicon/Millipore, Temecula, CA, USA), E‐cadherin (Cell Signaling Technology, Beverly, MA, USA), fibronectin (Sigma), HIF‐1α (BD Biosciences, San Jose, CA, USA), HIF‐1β (BD BiosciencesA), HIF‐2α (Novus Biologicals, Littleton, CO, USA), LOXL2 (R&D Systems, Minneapolis, MN, USA), vimentin (Sigma), and ZO‐1 (Invitrogen, Carlsbad, CA, USA).

### 2.3 *In vitro* cell migration

The cell migration ability was evaluated using a Transwell system (Corning Costar, Cambridge, MA, USA), which allows cells to migrate through a polycarbonate membrane (8‐μm pore size). Briefly, A549 and HCC827 cells were incubated under normoxia or hypoxia in growth medium for 36 h, followed by serum starvation overnight (12 h). Then, 4 × 10^4^ cells in serum‐free medium (200 μL) were seeded in the upper well, followed by continuous incubation for an additional 24 h in normoxic or hypoxic chambers. The lower compartment was filled with growth medium (600 μL) containing 10% FBS. Nonmigrated cells on the upper surface of the filter membrane were removed, and the migrated cells attached to the bottom surface of the filter membrane were fixed in 4% paraformaldehyde and stained with 0.1% crystal violet. The numbers of migrated cells were counted in five randomly selected fields under a microscope, and each assay was repeated in triplicate.

### 2.4 RNA‐seq library construction and data processing

After 72‐h incubation under normoxia or hypoxia in growth medium, total RNA isolation and library construction of RNA‐seq was performed as previously described(Qian *et al*., 2014). Initial sequence quality control metrics were calculated using fastqc (version v0.11.3). Next, filtering and data cleaning were performed to remove sequencing adaptors and reads of insufficient quality (low‐quality base (<5) rate > 50%). Mapping was performed using hisat (version 0.1.6‐beta) with the default parameters (Kim *et al*., 2015). Reference genome annotation files were downloaded from NCBI (GRCh37.p5). A human genome reference was constructed from UCSC (GRCh37/hg19) chromosomes 1–22, X, and Y and mitochondrial DNA. Alignment index files for hisat version 0.1.6‐beta were built from this reference using the HISAT‐build programs. Visualization tracks for mapped reads and gene coverage were generated using bedtools‐v2.24.0. Subsequent data processing, including that for gene expression levels and DEGs, was statistically analyzed via the method of Audic and Claverie (Audic and Claverie, 1997). Gene expression levels were calculated using the reads per kb per million reads (RPKM) method (Mortazavi *et al*., 2008). The RPKM value of each transcript was directly employed for comparing differences in gene expression among the samples. Significantly differentially expressed genes (DEGs) were identified based on a threshold false discovery rate (FDR) ≤ 0.001 and a fold change ≥ 2.

### 2.5 MeDIP‐seq and hMeDIP‐seq library construction and data processing

DNA isolation was performed after 72‐h incubation under normoxia or hypoxia in growth medium for the applied cell lines and MeDIP‐Seq and hMeDIP‐Seq libraries were constructed, as described in a previous study (Gao *et al*., 2013). The libraries were sequenced using the Illumina HiSeq analyzer, according to the manufacturer's instructions. From the raw fastq files, quality control was performed on the raw sequence data using in‐house scripts and FASTQC. After stringent quality control, 23 million paired reads were obtained from each sample on average. Alignment to hg19 was performed using bwa software (version 0.7.12, Cambridge, UK). After aligning the clean reads to the reference human genome (hg19), we randomly selected 15 million reads from each remaining dataset, leading to less noise and allowing us to obtain comparable total reads for each sample. We then used RPM values (reads assigned per million mapped reads) as a measure of the level of methylation and hydroxymethylation in a specific genomic region (Chavez *et al*., 2010). A Perl script was employed to calculate RPM by defining specified bin sizes across the genome. Differentially methylated regions (DMRs) and differentially hydroxyl‐methylated regions (DhMRs) were defined as differentially read‐enriched regions from two samples by viewing one of the samples as a control; thus, peak regions correspond to more enriched regions in the other sample. MACS‐1.4.2 was used to detect the differentially read‐enriched regions between the two samples with the following parameters: ‐f BED ‐g hs ‐s 100 ‐p 1e‐5 –wig (Zhang *et al*., 2008).

### 2.6 ChIP‐seq library construction and data processing

Chromatin immunoprecipitation (ChIP) was performed as previously described (Zhang *et al*., 2013). Briefly, cells were rinsed with room temperature PBS and cross‐linked by 1% formaldehyde following sonication by a BioruptorTM200 to generate chromatin fragments between 100 and 500 bp. The solubilized chromatin fragments were then immunoprecipitated with antibodies against H3K4me3 (CST 9751) and H3K27me3 (CST 9733), respectively. DNA from chromatin immunoprecipitation was used to construct sequencing libraries following the protocol provided by the Illumina TruSeq ChIP Sample Prep Set A and sequenced on Illumina Xten with PE 150 method. trimmomatic (version 0.38, Aachen, Germany) was used to filter out low‐quality reads. Clean reads were mapped to the human reference genome (hg19) by bwa (version 0.7.15), allowing up to two mismatches. samtools (version 1.3.1, Cambridge, UK) was used to remove potential PCR duplicates, and macs2 software (version 2.1.1.20160309, Boston, MA, USA) was used to call histone modification peaks by default parameters (bandwidth, 300 bp; model fold, 5, 50; *q* value, 0.05). Wig files produced by macs software were used for data visualization by igv (version 2.3.91, Cambridge, MA, UK). MAnorm was applied for differential analysis of histone modifications (Shao *et al*., 2012).

### 2.7 Functional enrichment and basic statistical analyses

Functional enrichment analysis for genes associated with differential epigenetic modifications was performed using WebGestalt (Wang *et al*., 2013), which is freely accessible online. integrated genome browser (version 2.3.51, Kannapolis, NC, USA) was employed to visualize the original read density. r (version 3.2.0, Vienna, Austria) was used to perform the basic statistical analyses and chart plotting.

### 2.8 Artificial neural network prediction

The neuralnet package (Fritsch and Guenther, 2010) in r (https://CRAN.R-project.org/package=neuralnet) was applied for artificial neural network analysis. Default parameters were used, except the argument of hidden was fitted as *h* = *c*(30,10).

### 2.9 Quantitative real‐time PCR

Total RNA was extracted using the TRIzol^®^ Reagent (Ambion, Austin, TX, USA) and then treated with RNase‐free DNase I (Thermo Scientific, Waltham, MA, USA) for 30 min. 1 μg of total RNA was reverse‐transcribed using Thermo Scientific RevertAid First Strand cDNA Synthesis Kit according to the manufacturer's instructions. Quantitative real‐time PCR (qRT–PCR) was performed using the Applied Biosystems StepOnePlus Real‐Time PCR System (Life Technologies, Carlsbad, CA, USA). Reverse transcription PCR primer sequences were listed in Table S9. The qRT–PCR for each sample was conducted with three technical replicates. *RPLP0* was used as a reference gene. The PCR was carried out under these conditions: 95 °C for 10 min, followed by 40 cycles of 95 °C for 15 s, 60 °C for 60 s. Relative expression levels for target genes were calculated via the 2^−∆∆CT^ method.

## 3 Results

### 3.1 Hypoxia induces EMT in NSCLC cell lines

**Figure 1:**
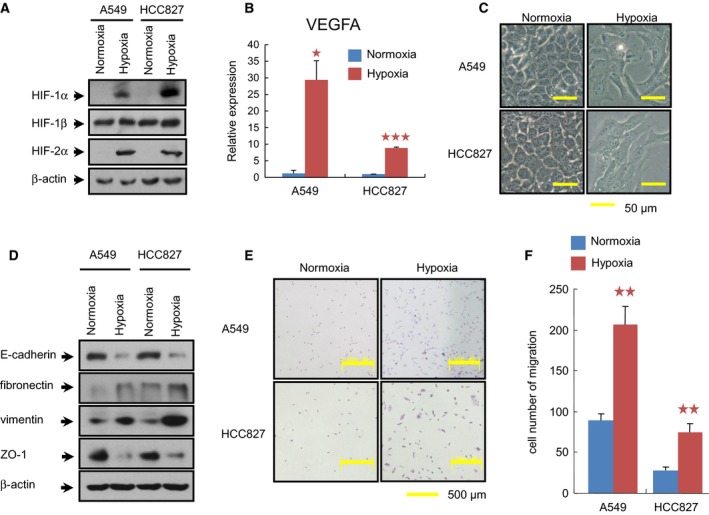
Induction of EMT by hypoxia in NSCLC cell lines. (A) Analysis of HIF proteins in cells exposed to normoxia (21% O_2_) or hypoxia (1% O_2_) for 6 h prior to preparation of whole‐cell extracts. (B) The effect of hypoxic exposure for 72 h on the transcriptional level of *VEGFA* was further evaluated by real‐time qPCR. The relative expression value for *VEGFA* mRNA was normalized on the basis of its *RPLP0* content. All the assays were performed in triplicate, and the data are shown as the mean values ± SEM. The asterisks denote significant differences (*P <* *0.05; ***P <* *0.001) within experiments, as determined by the Student's *t*‐test. (C) Morphological changes in cells treated with hypoxia for 72 h (Bars = 50 μm). Both A549 and HCC827 lost their epithelial honeycomb‐like morphology and obtained a spindle‐like shape. (D) Cellular protein levels of E‐cadherin, fibronectin, vimentin, and ZO‐1 affected by hypoxia for 72 h were determined by western blotting. β‐Actin was employed to ensure equal loading. E‐cadherin and ZO‐1 were down‐regulated, whereas fibronectin and vimentin were up‐regulated when cells were subjected to hypoxia. (E) As determined by transwell assay, the migratory cells under normoxia and hypoxia for 72 h were visualized by staining with crystal violet. (F) Quantification of these migratory cells determined by transwell assay in (E). Hypoxia treatment for 72 h clearly increased the migration of the cells of both cell lines. **P <* *0.01, as evaluated using the Student's *t*‐test.

Almost 85% of lung cancers are identified as NSCLC, among which adenocarcinoma is the most common histological subtype (Torre *et al*., 2016). Therefore, we collected two human NSCLC cell lines A549 and HCC827, which have been used extensively as an ideal *in vitro* model to study EMT, tumor hypoxia, and carcinogenesis (Chen *et al*., 2007; Feng *et al*., 2014). When exposed to hypoxia for 6 h, the proteins of HIF‐1α and HIF‐2α were accumulated in both A549 and HCC827 cells, whereas HIF‐1β was constitutively expressed (Fig. 1A). The activation of hypoxia signaling was further confirmed by assessing the mRNA level of *vascular endothelial growth factor A* (*VEGFA*) (Fig. 1B), a conventional target gene of HIF‐1α and HIF‐2α (Carroll and Ashcroft, 2006). After a prolonged treatment of hypoxia for 72 h, both HCC827 and A549 cells underwent phenotypic changes, as the cells lost their epithelial honeycomb‐like morphology and developed a spindle‐like shape (Fig. 1C). Along with these morphological alterations, the expression levels of the adherens junction protein E‐cadherin and the tight junction protein ZO‐1 were decreased, whereas the expression of the intermediate filament proteins fibronectin and vimentin was clearly up‐regulated (Fig. 1D). Because EMT is thought to promote tumor invasiveness and metastasis, we next investigated the impact of hypoxia on cell migration. Indeed, the ability of the cells to migrate was significantly increased when NSLCL cells were cultured in hypoxic conditions, compared with those under normoxia (Fig. 1E,F). Collectively, these results showed that hypoxic stimulus elicited a transition from epithelial to mesenchymal state in NSCLC cell lines.

### 3.2 RNA‐seq reveals a hypoxia‐mediated gene expression program in NSCLC cells

Next, we applied RNA sequencing (RNA‐seq) technology to characterize the transcriptional landscape of both A549 and HCC827 cells under normoxic and hypoxic conditions for 72 h. On average, 27.62 million raw paired‐end reads of 125 bp in length were generated in these cell lines. After removing low‐quality reads, 94.32% of the clean reads could be mapped to the human genome on average (hg19) (Table S1). For the majority of these mapped genes, the sequencing reads were randomly distributed in more than half of genic regions (Fig. S2A,B).

**Figure 2:**
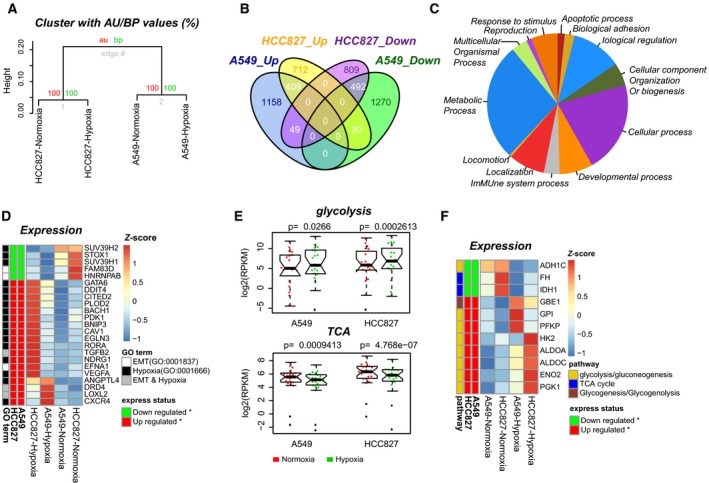
Extensive gene expression changes related to the hypoxia response, EMT and glycometabolism. (A) Hierarchical clustering of 16 620 commonly expressed genes between the two cell lines and the two cell states. (B) Venn diagram showing the shared and distinct DEGs between the two cell lines. (C) Classification of GO‐slim biological processes for the 901 DEGs shared between the two cell lines. (D) Heat map showing the 23 DEGs related to the GO terms of EMT and the response to hypoxia. Row annotation tracks indicate the expression status and GO terms of each DEG. (E) Gene expression changes related to glycolysis (up) and the TCA cycle (bottom) during epithelial (red) to mesenchymal (green) transition. The *P* value of a two‐tailed *t*‐test is given. (F) Heat map showing the 11 DEGs involved in glycometabolism. Row annotation tracks indicate the expression status and relative pathways of each DEG.

Typically, we identified 18 966 genes exhibiting at least one unique read, among which 16 620 genes were commonly expressed across the four samples (Fig. S2C). Hierarchical clustering analysis of the RPKMs of these commonly expressed genes revealed a larger difference between the two cell types than between the cell states under normoxic and hypoxic conditions, indicating distinct cell‐type‐specific transcription profiles (Fig. 2A). We then performed pairwise comparisons for the two cell lines, revealing 3398 and 2491 significantly altered genes in A549 and HCC827 cells exposed to hypoxia, respectively. Among these DEGs, 901 genes (409 up‐regulated and 492 down‐regulated) were shared between the two cell lines (Fig. 2B and Table S2). Using an online tool for the classification of gene function (http://www.pantherdb.org), we classified these 901 DEGs into functions in the ‘cellular process’ (279, 31.0%), ‘localization’ (110, 8%), and ‘response to stimulus’ (81, 9.0%) categories, among others (Fig. 2C). We suspected that many of the genes would be involved in the molecular pathways underlying hypoxia‐induced EMT; therefore, we cross‐matched these 901 DEGs with the 212 genes related to the gene ontology (GO) terms EMT (GO:0001837) and response to hypoxia (GO:0001666) and obtained 23 DEGs related to these two terms (Fig. 2D). Among these genes, *TGFB2*,* DRD4,* and *LOXL2* were shared between the EMT and hypoxia response terms and were all up‐regulated after hypoxia treatment (Table S3).

In addition to the DEGs classified in the hypoxia response and EMT pathways, we found that 39.6% (357) of the 901 DEGs were classified into ‘metabolic process’ functions. Further statistical testing indicated that these 357 genes were involved in both canonical glycolysis (7 genes) and glucose metabolic processes (12 genes) (Fig. S3 and Table S4). To clarify the gene expression of key metabolic pathways, we selected 118 key genes involved in the Kyoto Encyclopedia of Genes and Genomes (KEGG) pathways of glycolysis/gluconeogenesis (hsa00010, 67 genes), the citrate cycle (TCA cycle) (hsa00020, 30 genes), the pentose phosphate pathway (hsa00030, 29 genes), and glycogenesis/glycogenolysis (hsa00500, 12 genes). Comparison of the two cell lines under normoxic and hypoxic conditions showed that hypoxia notably activated the glycolysis pathway and significantly repressed the TCA cycle (Fig. 2E). 11 genes involved in glycol‐metabolism were shared between these two cell lines, thus representing key candidate genes in response to hypoxia (Fig. 2F).

### 3.3 Epigenome‐wide mapping illustrates a unique outlook of hypoxic NSCLC cells

**Figure 3:**
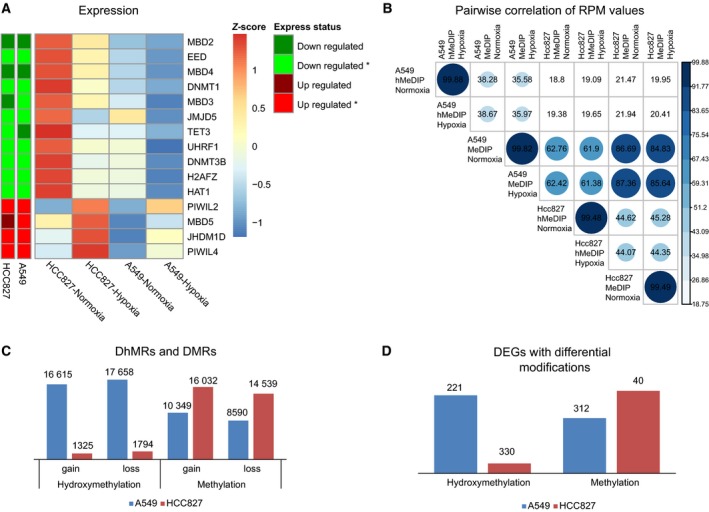
Differences in epigenetic modifications associated with gene expression in NSCLC cells. (A) The expression of 15 genes associated with epigenetics. Row annotation tracks indicate the expression status of each gene in the two cell lines. *Mean significant changes in expression. (B) Pairwise correlations of epigenetic modification levels in all samples. The RPM values per 2 kb of the genome were used to calculate Pearson's correlations. (C) Numbers of DMRs and DhMRs in the two cell lines. (D) Numbers of DEGs harboring differential epigenetic modifications in each cell line.

We hypothesized that oxygen levels may affect functional proteins containing 2‐oxoglutarate‐ and iron(II)‐dependent dioxygenase (2OGFeDO) domains, especially proteins in the TET and methyl binding protein domain (MBD) families (Iyer *et al*., 2009), resulting in transcriptional regulation of the DNA methyltransferase (DNMT) family as well as other factors involved in DNA methylation. Therefore, we further examined the DNA methylation‐related genes, including *DNMTs*,* TETs*,* MBDs,* and nucleosome‐ or chromatin‐related genes (Ooi *et al*., 2009), among the 901 DEGs identified above (Table S2). As a result, we found that *TET3*,* UHRF1*,* DNMT3B*,* MBD2*,* MBD3,* and *MBD4* were all significantly down‐regulated after hypoxia exposure in both cell lines. In contrast, the chromatin‐related genes *PIWIL2* and *PIWIL4* were significantly up‐regulated (Fig. 3A). Interestingly, the expression levels of other members of the DNMT and TET families, that is, DNMT1, DNMT3A, and Tet1/2, were not altered. We then validated the relative expression of *DNMT3B* and *TET3* in A549, HCC827, and three other NSCLC cell lines (NCI‐H838, NCI‐H1437, and NCI‐H1975), and the results also showed down‐regulation of those genes (Fig. S4). As the functions of these enzymes are tightly regulated for establishing, maintaining, and modifying DNA methylation patterns, these results might suggest that the hypoxia‐induced alterations of DNA methylome were mainly mediated by DNMT3B and TET3.

We subsequently applied the methylated DNA immunoprecipitation (MeDIP‐seq) and hydroxymethylated DNA immunoprecipitation (hMeDIP‐seq) techniques to determine the genomewide profiles of DNA methylation and hydroxymethylation, respectively. The results regarding data generation and quality control for these sequencing experiments are displayed in Table S5 and Fig. S5. Initially, we investigated the overall 5mC and 5hmC patterns of each library. Our results showed that the distribution of 5mC and 5hmC was considerably divergent, although many of the modified regions overlapped (Figs S5 and S6). We then applied Pearson correlations of reads per million (RPM) values to infer the dynamics of DNA modifications. The difference between the overall 5mC and 5hmC levels in A549 and HCC827 cells under normoxia versus hypoxia was small. Instead, greater dissimilarity between overall 5mC and 5hmC levels as well as between the two cell lines was observed (Fig. 3B). Furthermore, analysis of modification levels against GC contents showed a similar pattern of the 5mC distribution between the two cell lines (Fig. S7A). In comparison with methylation, the distribution of 5hmC was considerably different, as 5hmC was abundant in regions of low CpG content in HCC827 cells, whereas 5hmCs were more enriched in regions with a high GC content in A549 cells (Fig. S7B). Further, we identified many more DhMRs, but slightly fewer DMRs in A549 cells compared with HCC827 cells (Fig. 3C). Accordingly, more DhMR‐containing but less DMR‐containing DEGs out of the 901 common DEGs were revealed (Fig. 3D). This difference could be explained by the distinct stages of cellular differentiation in these two cell lines, as pluripotent cells show more 5hmCs distributed in low‐GC regions (Yu *et al*., 2012).

In addition, we applied ChIP‐seq to investigate the dynamic changes in genomewide histone modifications of H3K4me3 and H3K27me3, representing the active and repressive markers, respectively. As a result, a higher global divergence of H3K27me3 in comparison with H3K4me3 was observed between the hypoxia and normoxia states of the two cell lines (Fig. S8), in accordance with the significantly differential regions we identified (Table S6).

### 3.4 HIF target genes potentially regulated by hypoxia via DNA modification

**Figure 4:**
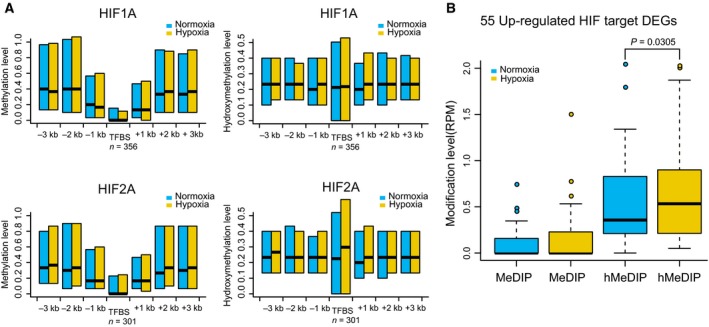
Hypoxia‐induced factor binding sites and target genes indicate a variety of cellular responses to hypoxia. (A) Distribution of methylation and hydroxymethylation levels at 3 kb upstream and downstream of HIF binding sites. (B) 55 up‐regulated HIF target DEGs show significantly increased hydroxymethylation. The P value of a paired Wilcox test is given.

Hypoxia‐inducible factors stimulate transcription via binding to HREs in the promoters of target genes, which contain core 5′‐[AG]CGTG‐3′ sequences (Pollard *et al*., 2008). Previous studies have indicated that hypomethylation of HREs is required for HIFs to bind to their target genes, although it is unclear whether this is the only regulatory mechanism (Horiuchi *et al*., 2012; Kitamoto *et al*., 2012). As shown in Fig. 1A, HIF‐1α and HIF‐2α were accumulated in response to hypoxia exposure in both A549 and HCC827 cells, whereas HIF‐1β was constitutively expressed. We therefore studied 438 target genes of HIF‐1α (323 genes) and HIF‐2α (267 genes) that were revealed in a previous study via high‐resolution ChIP‐seq technology (Schodel *et al*., 2011). The results clearly showed that HIF binding regions were generally hypomethylated in comparison with flanking regions. In contrast, highly variable hydroxymethylation levels were observed in HIF binding regions, which might suggest dynamic regulation of methylation around HIF binding regions in the cell population (Fig. 4A). Among these candidate target genes of HIFs, 55 DEGs were identified as significantly up‐regulated in our transcriptome analyses of the two cell lines after hypoxia (Table S3). We further examined the methylation and hydroxymethylation status of the binding regions of these 55 genes. Interestingly, we observed significantly greater hydroxymethylation after hypoxia based on intragroup comparison of these genes, whereas for methylation, a hypomethylated status was generally maintained, with no significant difference being observed (Fig. 4B). Considering that 5hmC is the intermediate of demethylation, these results suggest that a large population of cells were in the process of undergoing demethylation in response to hypoxia, thereby enabling HIF binding.

### 3.5 Selection and validation of a candidate gene set as biomarker of lung cancer metastasis

We propose a rationale that the epigenetic and transcriptional alterations induced by hypoxia in NSCLC cell lines were comparable to those in the progression and metastasis of NSCLC. Based on this reasoning, we next selected a set of genes as candidates of metastasis biomarkers of lung cancer by integrating epigenomic, transcriptomic, and HIF target analyses mentioned above. We thereby obtained 51 genes that contained genes with divergent promoter DNA modifications across the two cell lines (Table S3 and Fig. S8), genes in the EMT and hypoxia response GO pathways, genes involved in glycometabolism and epigenetics‐related genes (Table S3). We reasoned that the gene expression profiles of hypoxic NSCLC cell lines could be comparable to those in invasive lung adenocarcinomas. Therefore, we further compared the expression data for 51 genes between hypoxic NSCLC cells and a dataset that contains microarray gene expression data of microdissected tumor samples from 17 cases of noninvasive lung adenocarcinoma and 23 cases of adenocarcinoma with mixed‐subtype invasive tumors [Gene Expression Omnibus (GEO) accession code GSE27716]. As a result, we identified 29 genes that displayed the same trend of expression changes (Table S7).

To validate whether these genes preferentially presented a unique expression signature of hypoxic NSCLC cells, we then selected three representative key genes (*LOXL2*,* VEGFA,* and *ANGPTL4*) that were known to be responsible for hypoxia, EMT, and glycometabolism and applied quantitative real‐time PCR to validate their mRNA levels in three independent NSCLC cell lines (NCI‐H838, NCI‐H1437, and NCI‐H1975), in addition to A549 and HCC827. Furthermore, protein expression of ANGPTL4 and LOXL2 was confirmed by western blotting in these NSCLC cell lines. Consistently, these genes were mostly up‐regulated by hypoxia except *LOXL2* in NCI‐H1437 (Fig. S9), despite these NSCLC cells possessing varied mutational profiles (See Materials and methods).

**Figure 5:**
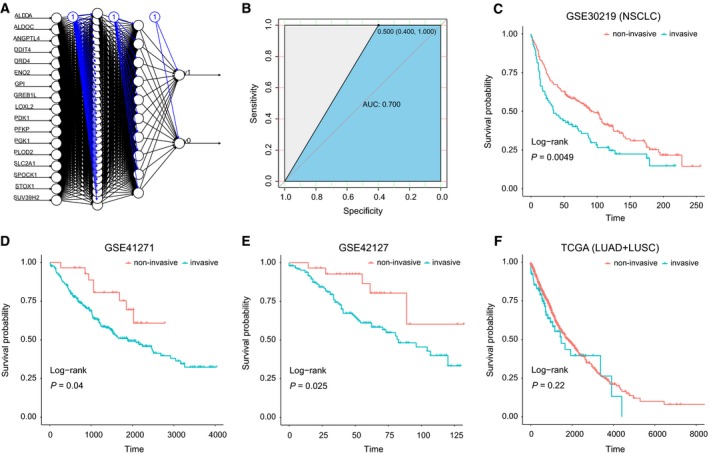
Training and validation of the 17‐gene panel by gene expression datasets of NSCLC patients. (A) Schematic display of the artificial neural network analysis using the 17‐gene signature; (B) the receiver operating characteristic curve (ROC) analysis of the 17‐gene signature in the training dataset; an ANN algorithm was applied to classify the NSCLC samples from GEO datasets, including (C) GSE30219 (*n* = 149), (D) GSE41271 (*n* = 275), and (E) GSE42127 (*n* = 176). A dataset containing 21 SCLC samples were also analyzed (F). Survival analyses are applied to the two classified groups, as indicated in the column annotation (red and blue bars). Wald test *p* values are given for each survival analysis.

Encouraged by these results, we then aimed to develop a predictive gene panel for metastasis of NSCLC, using randomly selected 30 samples from the GSE27716 dataset as a discovery set and the rest 10 samples as a test set. We applied an algorithm based on artificial neural network (ANN) (Fritsch and Guenther, 2010) (Fig. 5A) and mainly tested three combinations of the 29 genes, including the up‐regulated (17), down‐regulated (12), and total genes (Table S7). As a result, we found that the 17 up‐regulated gene panel displayed better accuracy than the total or the down‐regulated gene set (data not shown), which can achieve a prediction accuracy of 70% and sensitivity of 100% (Fig. 5B). Probably due to limited sample size, the specificity rate is 40%.

Further, as metastasis is responsible for more than 90% of deaths among cancer patient with solid tumors (Gupta and Massague, 2006), we assumed that these genes could reflect the survival time of patients. We then downloaded four independent datasets containing gene expression data and survival rate information of NSCLC patients, including three GEO datasets (GSE30219, GSE42127, and GSE41271) and a combined TCGA dataset (TCGA_LUSC + TCGA_LUAD) (Table S8). We applied the same ANN algorithm to predict the patients into two groups and analyzed their survival rates for the two predicted patient groups. As a result, three datasets of NSCLC showed significant differences in survival time between the two classified groups (*P* values are 0.0049, 0.04, and 0.025, respectively) (Fig. 5C–E). Though the combined TCGA NSCLC dataset was not showing significance, these results indicated for the efficacy of this 17‐gene signature to distinguish the high or low risk of survival in patients with NSCLC. Furthermore, we also examined another three datasets containing transcriptome data of lung small cell carcinoma (SCC), hepatocellular carcinoma (LIHC), and stomach adenocarcinoma (STAD). No significant difference was observed between the two predicted patient groups for their survival rates (Fig. S10). These results might indicate the tumor‐specific potential for this 17‐gene panel as a tool to identify metastatic‐prone NSCLC tumors. However, considering the technology bias and small sample size among these datasets, further study with larger sample size is needed to establish a solid conclusion whether the 17‐gene panel is highly specific to NSCLC.

## 4 Discussion

The exceptionally high mortality of lung cancer can be investigated by treatment resistance and progressive metastasis. There is now a pressing need to determine the malignant status of cancer lesions and the prognostic significance of novel biomarkers, as these tools will enable the clinical development of personalized cancer therapies that will ultimately improve patient outcomes. The aims of this study were to first characterize unique epigenomic and transcriptomic signatures in NSCLC cells under hypoxia and then to identify a gene panel that could serve as potential biomarkers with prognostic implications in the clinic. Previous studies have indicated that the molecular program of metastasis is already present in the bulk of some primary cancers at the time of diagnosis (Ramaswamy *et al*., 2003). Here, we have proposed a molecular program for metastasis, involving both gene expression and epigenetic modifications, which were inferred from hypoxic NSCLC cell lines, as hypoxia is a vital feature in the tumor microenvironment that can drive EMT and metastasis.

We applied high‐throughput sequencing technologies for analyzing RNA populations (RNA‐Seq), DNA modifications (MeDIP‐seq and hMeDIP‐seq), and histone modifications (H3K4me3 and H3K27me3). These analyses indicated that oxygen, as a major environmental factor, can indeed cause extensive alterations of gene expression. Thus, oxygen can ultimately regulate cellular metabolism and cell‐fate decisions. Importantly, in addition to the genes involved in the classical pathways of hypoxia and EMT, a considerable proportion of the identified genes were involved in glucose metabolism. The glycolysis pathway was significantly activated, and the TCA pathway was significantly repressed after hypoxic stimulus (Figs 2E and S3). These findings are in agreement with the view of the functional importance of glycolysis in cancer (Altenberg and Greulich, 2004). Furthermore, previous studies revealed that several epigenetic enzymes are dependent on oxygen levels in the cell, and these epigenetic enzymes appear to play HIF‐independent roles in regulating glycometabolism and cell proliferation (Kaelin, 2011), indicating potential interactions among epigenetic modulation, oxygen‐dependent molecular pathways, and regulation of glycometabolism. As expected, we found that hypoxia regulated the expression levels of several key DNA modification enzymes and consequently induced globally dynamic changes in genomewide DNA methylation, hydroxymethylation, as well as histone modifications. Many DEGs also displayed significant variations in DNA modifications, including key HIF target genes (Fig. 4B), suggesting that DNA modification may be involved in cellular responses to hypoxia. Based on these results, we identified a matrix of gene signatures in hypoxic NSCLC cell lines.

To identify a gene panel that could be used as biomarkers of the metastasis program in primary NSCLC, we tested several machine‐learning algorithms in a dataset (GSE27716) containing gene expression data of both invasive and noninvasive lung adenocarcinoma, using three fourths of the samples as the discovery set and the rest as the test set of samples. Ultimately, we obtained a 17‐gene panel that could be used as a potential molecular signature for prediction of metastasis and prognostic stratification in patients with NSCLC (Fig. 5C–E). Interestingly, all these 17 genes were significantly up‐regulated upon hypoxia exposure, while the gene set with down‐regulated genes were not applicable. Among these up‐regulated genes, *ANGPTL4* was strongly regulated by epigenetic mechanisms as we observed alterations of both DNA and histone modifications upon hypoxia exposure across the two cell lines (data not shown). *ANGPTL4* was previously reported to be induced by hypoxia and correlate with NSCLC progression (Zhu *et al*., 2016). More relevantly, 10 out of these 17 genes are HIF‐targeted genes, in agreement with increased HIF expression in response to hypoxia exposure. Six genes, including phosphoglycerate kinase (*PGK1*), enolase (*ENO2*), and four of these HIF‐targeted genes (glucose‐6‐phosphate isomerase (*GPI*), phosphofructokinase (*PFKP*), aldolase (*ALDOA* and *ALDOC*)) are involved in glycolysis, consistent with the well‐accepted concept of up‐regulation of glycolysis in cancer (Semenza, 2010). Therefore, these six genes represent the key regulators controlling the metabolic pathways of lung cancer cells, leading to increased anaerobic glycolysis in response to hypoxia. In addition, the up‐regulated HIF‐1 target gene pyruvate dehydrogenase kinase‐1 (*PDK1*), together with the up‐regulated cell‐surface glucose transporter *SLC2A1* (*GLUT1*), plays a key role in blocking the aerobic TCA cycle by inhibiting the oxidative decarboxylation of pyruvate (Gagliardi *et al*., 2015; Wigfield *et al*., 2008). Previous work also revealed a role of *SLC2A1* in the prognosis of metastasis in lung cancer (Buffa *et al*., 2010). On the other hand, the down‐regulated *ADH1C* redirects pyruvate metabolism to lactate production, as it suppresses the pathway of ethanol production. Accumulation and secretion of lactate have been found to represent a pivotal early event that is correlated with cell migration and tissue invasion in cancer (Goodwin *et al*., 2014; Hirschhaeuser *et al*., 2011).

One major shortage of the current study is the small sample size we obtained for the algorithm training. Due to difficulties of getting metastatic samples, such dataset is very rare now in the public databases. Despite that, we identified a 17‐gene panel as potential biomarkers for precise prediction of metastasis and prognosis in patients with NSCLC. Future studies are required to further improve the efficacy and warrant its potential usefulness in the diagnosis, prognosis, and epidemiology of lung cancer.

## 5 Conclusion

We identified a 17 gene panel for NSCLC prognosis through integrative epigenomic–transcriptomic analyses of hypoxia‐induced epithelial–mesenchymal transition. Our study may be suitable as a basis for further selection of gene signature to potentially guide prognostic stratification in patients with NSCLC.

## Conflict of interest

The authors declare no conflict of interest.

## Author contributions

FG and ZS designed experiments and interpreted data. YZ conducted bioinformatic and statistical analyses with help from NC. YC established the EMT cell model and performed western blots. JW performed MeDIP/hMeDIP and transcriptome sequencing library construction. WF, JZ, and YL contributed to samples collection; and RQ and XY performed quantitative RT–PCR validation experiments. FG wrote the manuscript with help from YC for revision. All authors have read and approved the manuscript for publication.

## Supporting information


**Fig. S1.** Workflow chart of data generation and analysis.
**Fig. S2.** Quality control for RNA sequencing data.
**Fig. S3.** GO enrichment analysis of 357 DEGs associated with metabolic processes.
**Fig. S4.** *DNMT3B* and *TET3* mRNA expression levels in NSCLC cell lines quantitated by real time‐PCR.
**Fig. S5.** Coverage and sequencing depth for MeDIP‐seq and hMeDIP‐seq.
**Fig. S6.** Distribution of epigenetic modifications in each genomic element.
**Fig. S7.** Genome‐wide comparison of epigenetic modification levels against GC contents.
**Fig. S8.** Scatter plots of the average ChIP signals of each histone modification (H3K4me3 and H3K27me3) between hypoxia and normoxia cells.
**Fig. S9.** The validation of expression changes of three genes (*ANGPTL4*,* LOXL2* and *VEGFA*) in five cell lines.
**Fig. S10.** The survival analyses for four datasets of TCGA.

**Table S1.** Summary of RNA‐seq data production and alignment result.
**Table S2.** Summary of 901 Differentially expressed genes shared in two cell lines.
**Table S3.** Differentially expressed genes with multiple characteristics.
**Table S4.** GO enrichment analysis of 357 Differentially expressed genes involved with metabolic process.
**Table S5.** Summary of MeDIP‐seq and hMeDIP‐seq data production and alignment result.
**Table S6.** Summary of significantly differential regions of histone modifications in the two cell lines.
**Table S7.** 29 candidate genes with multiple characteristics.
**Table S8.** Public datasets applied.
**Table S9.** Primer sequences for RT‐PCR.
