# Supplementary material for: A 17 gene panel for non‐small‐cell lung cancer prognosis identified through integrative epigenomic‐transcriptomic analyses of hypoxia‐induced epithelial–mesenchymal transition
Source: Mol Oncol. 2019 May 29;13(7):1490–502. doi: 10.1002/1878-0261.12491 (PMC6599842; doi:10.1002/1878-0261.12491)
Supplement: Supplementary file 1 — Fig. S1. Workflow chart of data generation and analysis. Fig. S2. Quality control for RNA sequencing data. Fig. S3. GO enrichment analysis of 357 DEGs associated with metabolic processes. Fig. S4. DNMT3B and TET3 mRNA expression levels in NSCLC cell lines quantitated by real time‐PCR. Fig. S5. Coverage and sequencing depth for MeDIP‐seq and hMeDIP‐seq. Fig. S6. Distribution of epigenetic modifications in each genomic element. Fig. S7. Genome‐wide comparison of epigenetic modification levels against GC contents. Fig. S8. Scatter plots of the average ChIP signals of each histone modification (H3K4me3 and H3K27me3) between hypoxia and normoxia cells. Fig. S9. The validation of expression changes of three genes (ANGPTL4, LOXL2 and VEGFA) in five cell lines. Fig. S10. The survival analyses for four datasets of TCGA. [file MOL2-13-1490-s001.doc]

# Integrative epigenomic-transcriptomic analyses of hypoxia-induced epithelial–mesenchymal transition reveal a 17-gene panel associated with prognosis in non-small cell lung cancer

Yue-Lei Chen^4#^, Yihe Zhang^3#^, Junwen Wang^5^, Na Chen^3^, Weiying Fang^4^, Jianing Zhong^1^, Yi Liu^4^, Rui Qin^3^, Xinxin Yu^3^, Zhongsheng Sun^2*^, Fei Gao^1,3*^

1 Institute of Genomic Medicine, Wenzhou Medical University, Wenzhou 325000, China

2 Beijing Institutes of Life Sciences, Chinese Academy of Sciences, Beijing 100101, China

3 Agricultural Genomics Institute at Shenzhen, Chinese Academy of Agricultural Sciences, Shenzhen 518120, China

4 Stem Cell Bank/Stem Cell Core Facility, Institute of Biochemistry and Cell Biology, Shanghai Institutes for Biological Sciences, Chinese Academy of Sciences, Shanghai 200031, China

5 E-GENE Co., Ltd., Shenzhen 518118, China

^#^ These authors contributed equally to this work.

* Correspondence:

Zhongsheng Sun, Beichen West Road, Chao Yang District, Beijing 100101, China. Tel: +10 64864959; E-mail: [sunzs@mail.biols.ac.cn](mailto:sunzs@mail.biols.ac.cn);

Fei Gao, 7 Pengfei Road, Dapeng New District, Shenzhen 518120, China. Tel: +86 755 23251432; E-mail: [flys828@gmail.com](mailto:flys828@gmail.com);


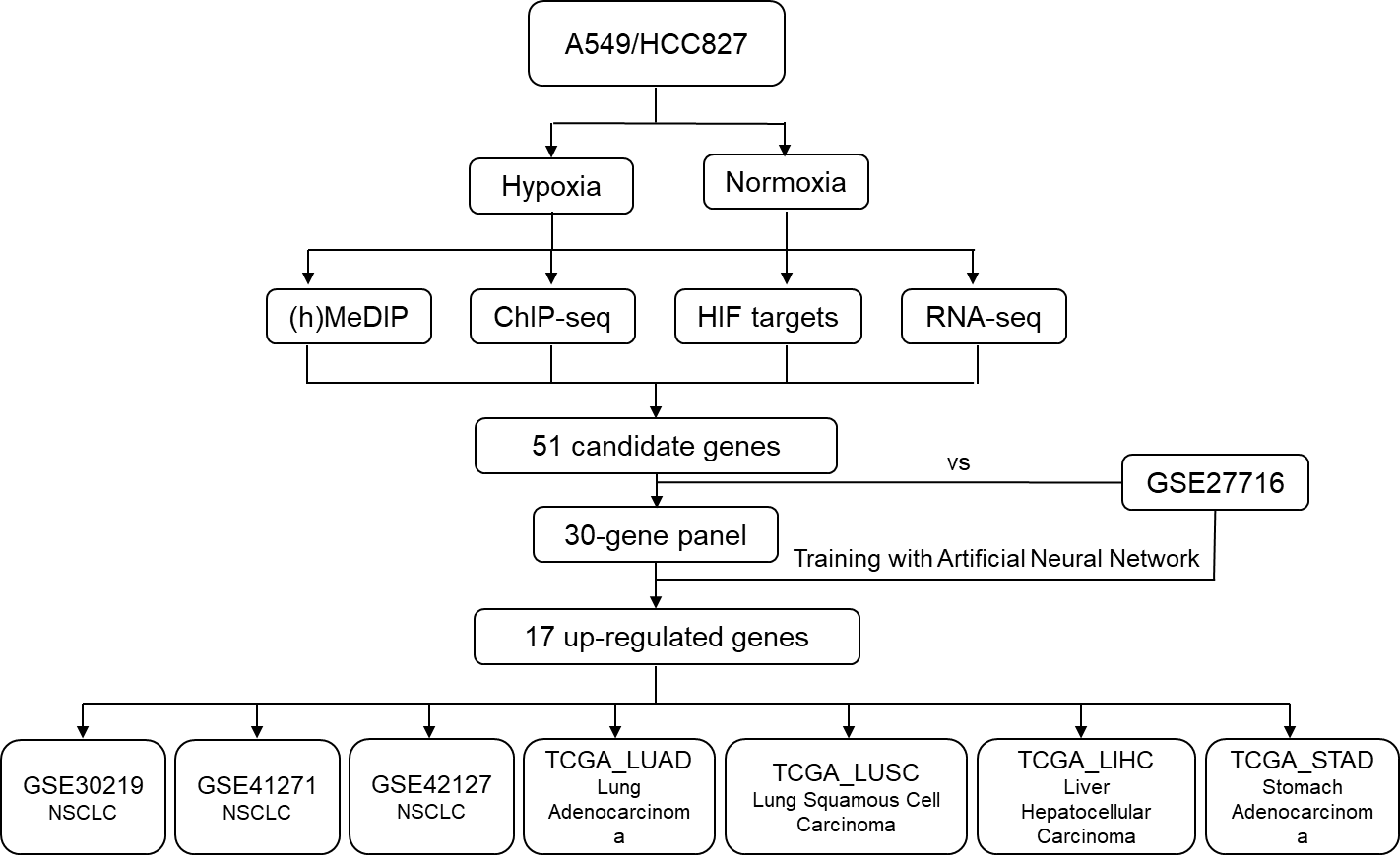


**Supplementary Figure 1. Workflow chart of data generation and analysis.**


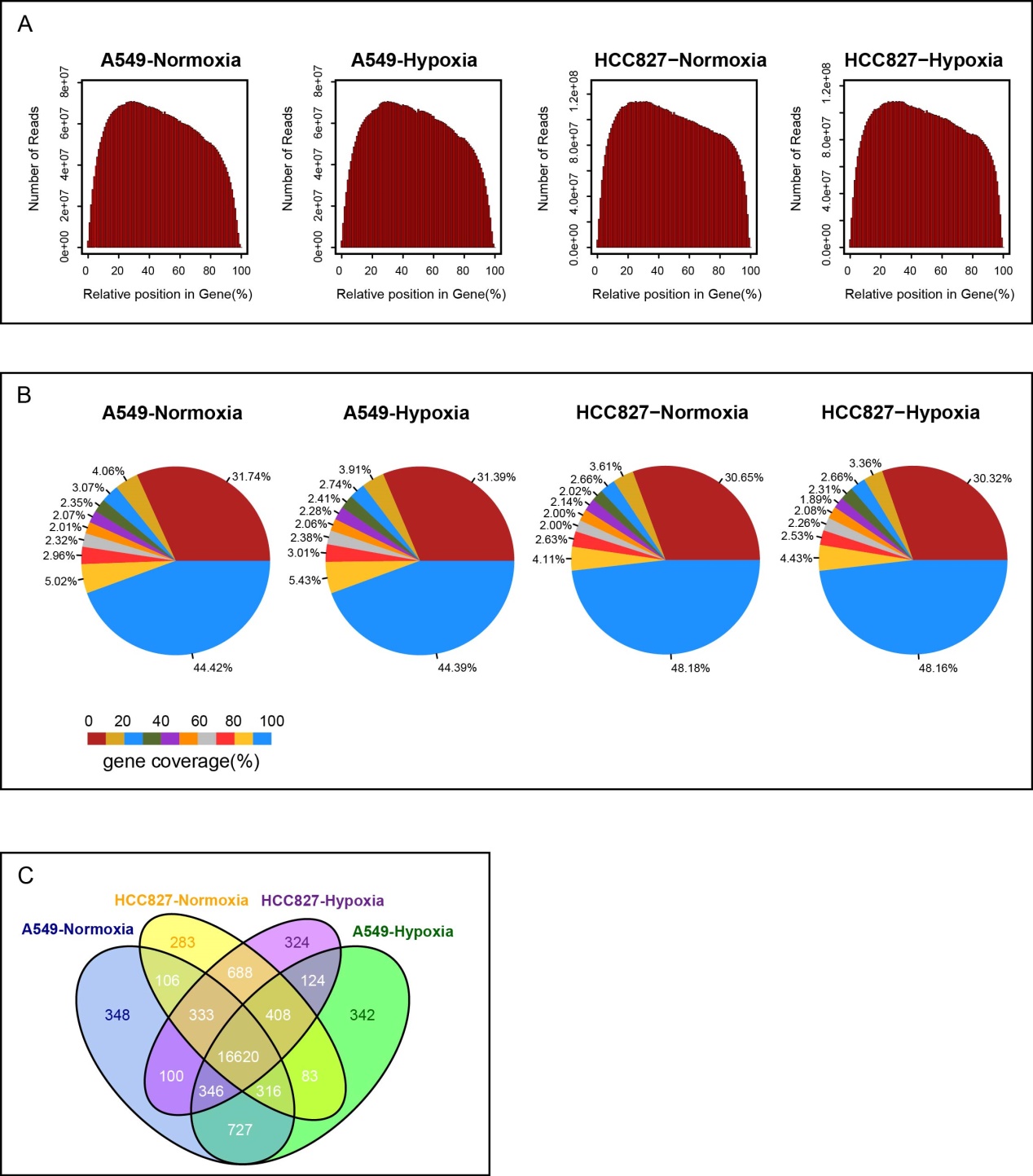


**Supplementary Figure 2. Quality control for RNA sequencing data.** (A) Total read distribution across all transcripts. Transcripts with a length > 200 bp were split into 100 bins, and the total number of reads was calculated for each bin. (B) Coverage distribution of all transcripts. Transcript coverage is divided into 10 different grades with 10% spacing. For each grade of coverage, we calculated the percentage of the covered gene. (C) Venn diagram showing the shared and specific covered genes across all samples.


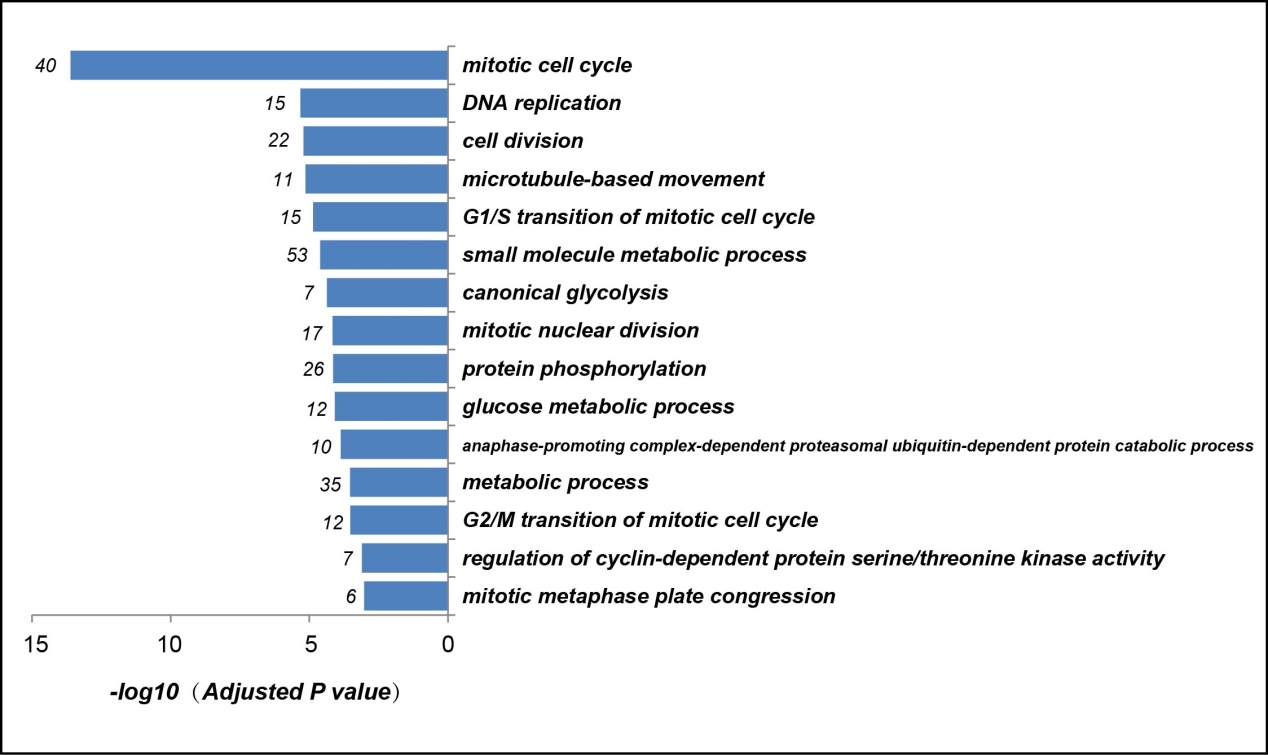


**Supplementary Figure 3. GO enrichment analysis of 357 DEGs associated with metabolic processes.** Enriched GO terms with an adjust p value < 0.001 are shown.


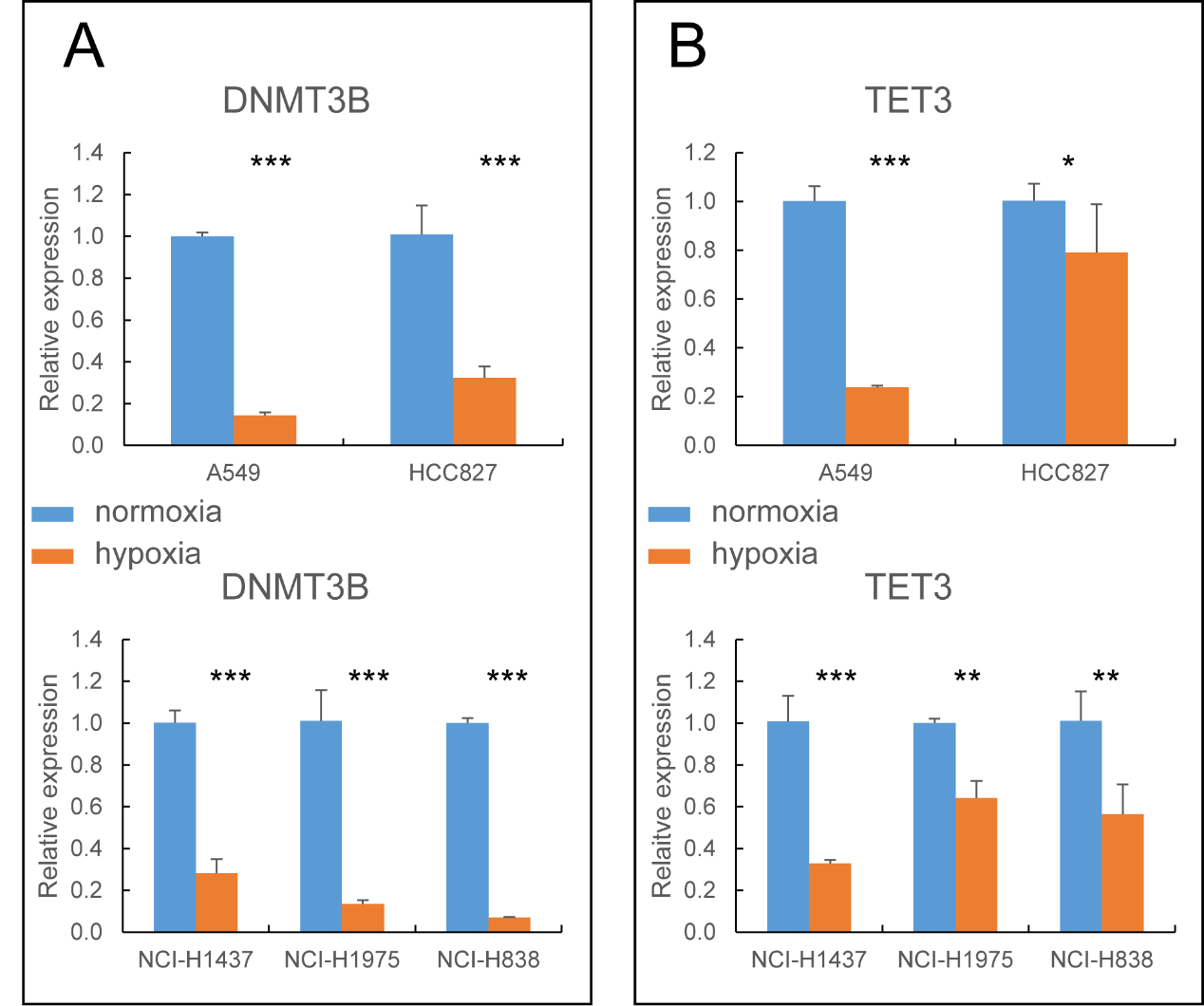


**Supplementary Figure 4. *DNMT3B* and *TET3* mRNA expression levels in NSCLC cell lines quantitated by real time-PCR.** (A) gene *DNMT3B* mRNA levels in cell line A549, HCC827 (top) and NCI-H1437, NCI-H1975 and NCI-H838 (bottom). (B) gene *TET3*. All the assays were performed in triplicate, and the data are shown as the mean values ± SEM. The asterisks denote significant differences (*, *P*<0.05; ***, *P*<0.001) within experiments, as determined by the Student’s *t*-test.


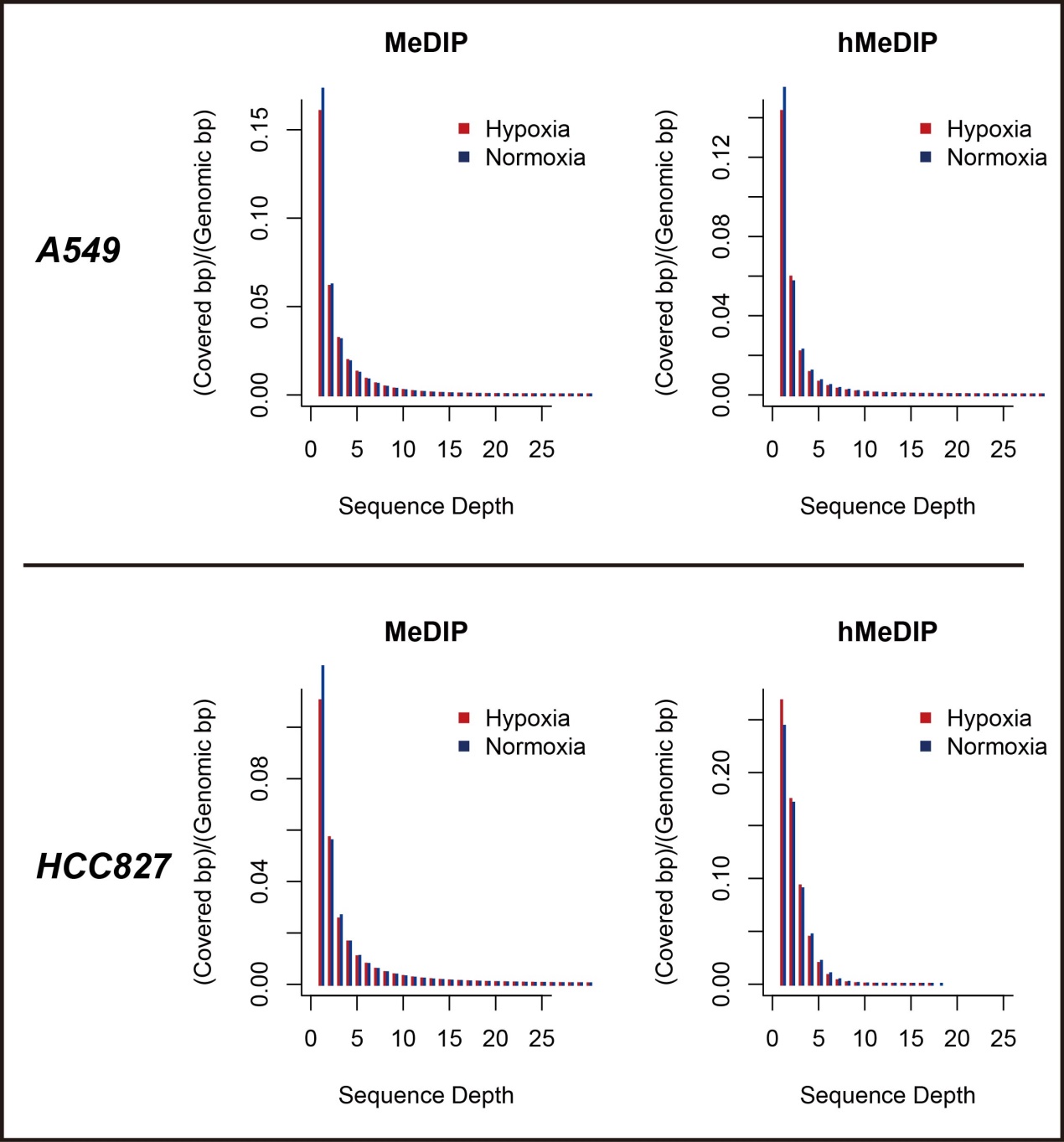


**Supplementary Figure 5. Coverage and sequencing depth for MeDIP-seq and hMeDIP-seq.** The bars indicate the genomic coverage for the specified sequencing depth.


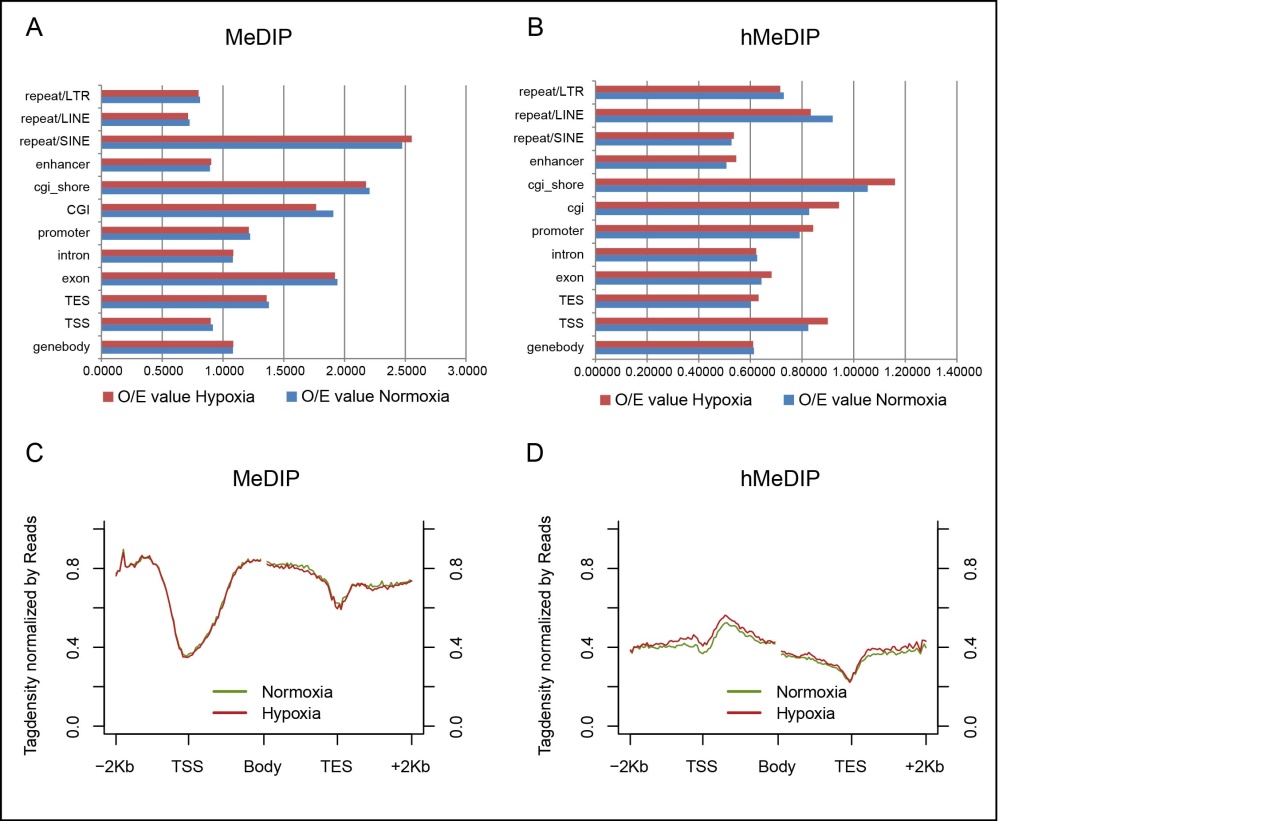


**Supplementary Figure 6. Distribution of epigenetic modifications in each genomic element.** (A) Enrichment of DNA methylation modifications identified, with genomic features, CGIs and the neighboring context, and repetitive sequences. CGI shore, 0–2 kb from the CGI; SINE, short interspersed nuclear element; LINE, long interspersed nuclear element; LTR, long terminal repeat. An O/E value >1 indicates enrichment for the specified element. (B) Enrichment of DNA hydroxymethylation modifications identified, with genomic features, CGIs and the neighboring context, and repetitive sequences. The average methylation (C) and hydroxymethylation (D) modifications within 2 kb of the TSS and TES for all genes are shown here.


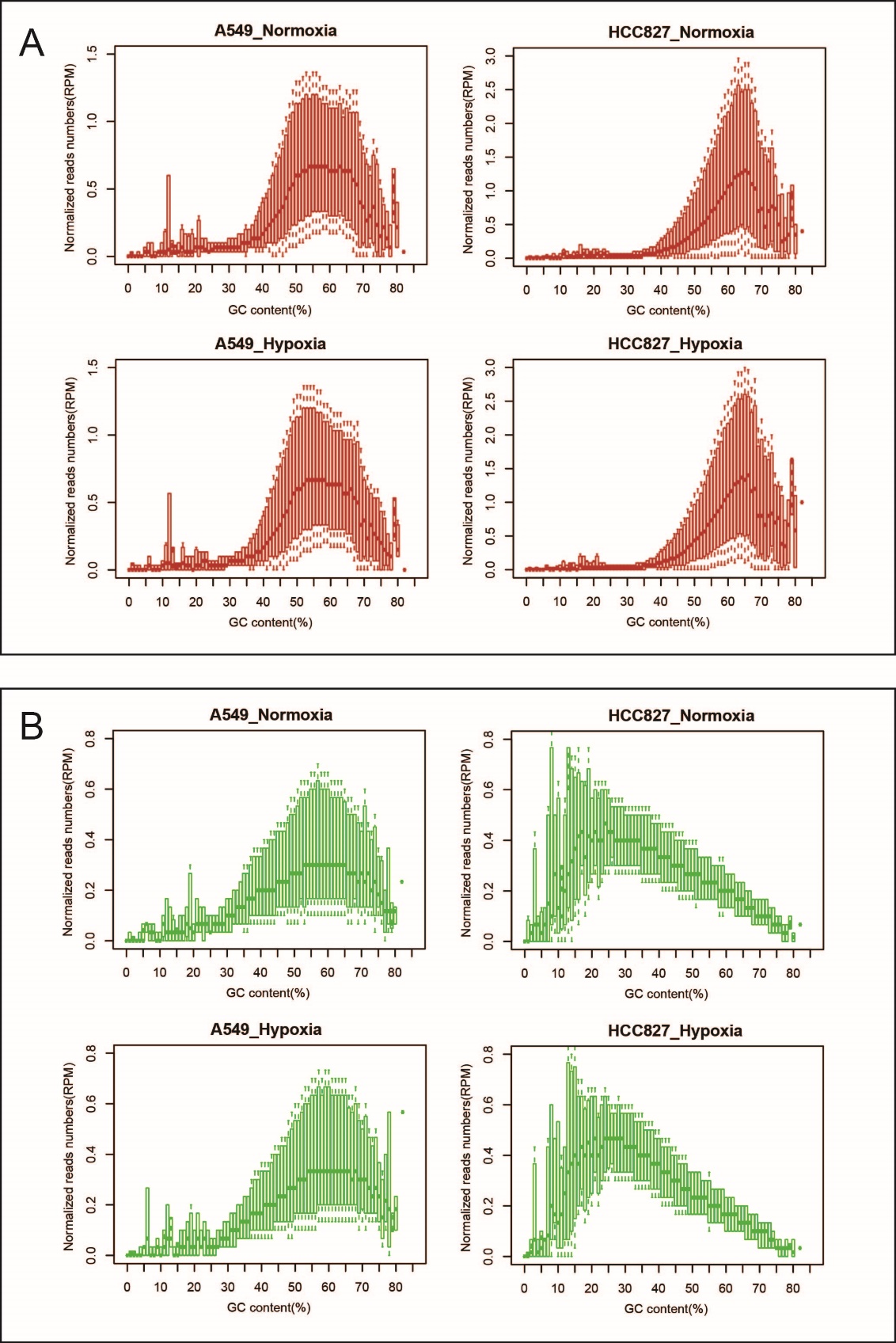


**Supplementary Figure 7. Genome-wide comparison of epigenetic modification levels against GC contents.** (A) Levels of methylation modifications against GC contents genome-wide. (B) levels of hydroxymethylation modifications against GC contents genome-wide. The entire genome was split into 2 kb-length bins, and, the GC content and RPM value were calculated for each bin.


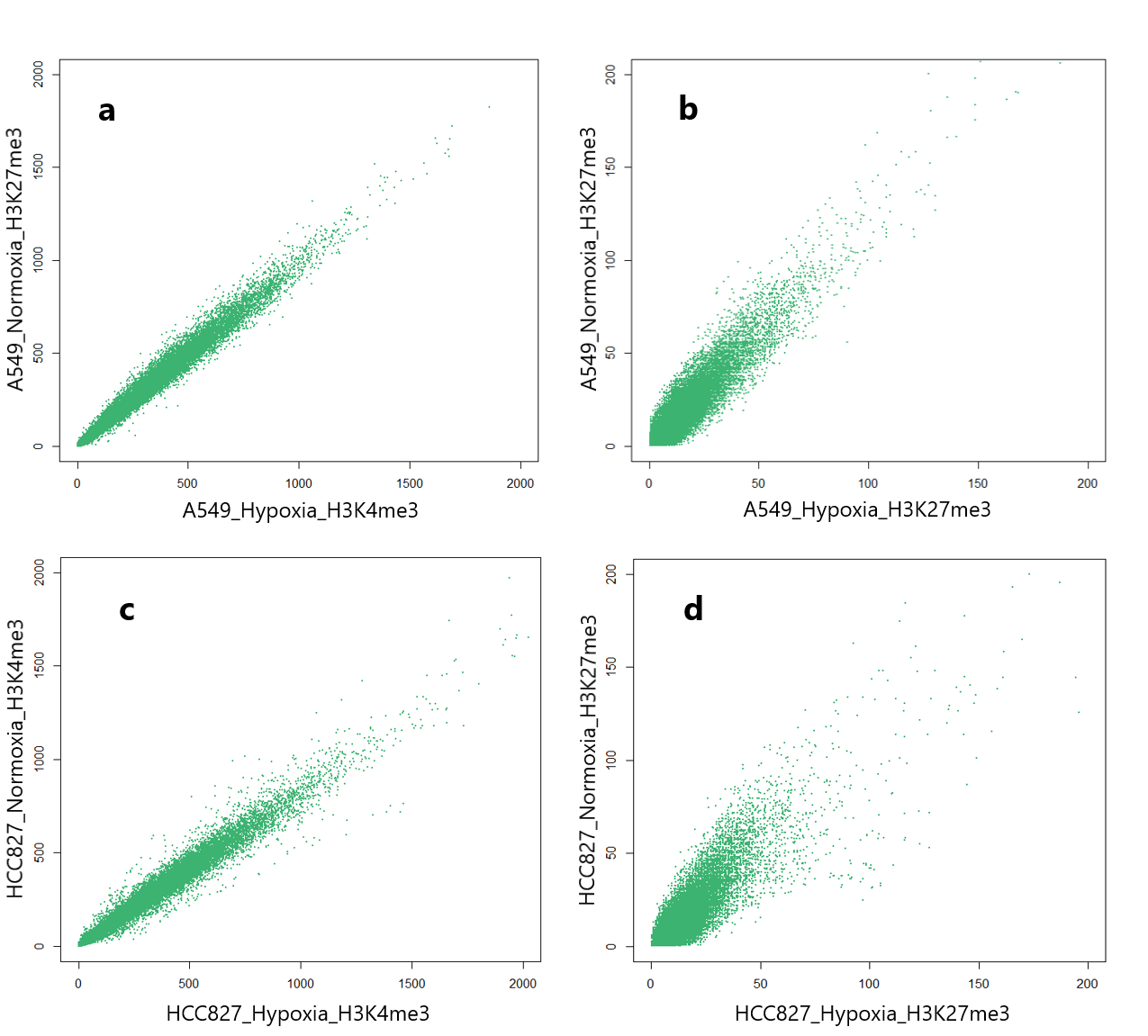


**Supplementary Figure 8.** Scatter plots of the average ChIP signals of each histone modification (H3K4me3 and H3K27me3) between hypoxia and normoxia cells.


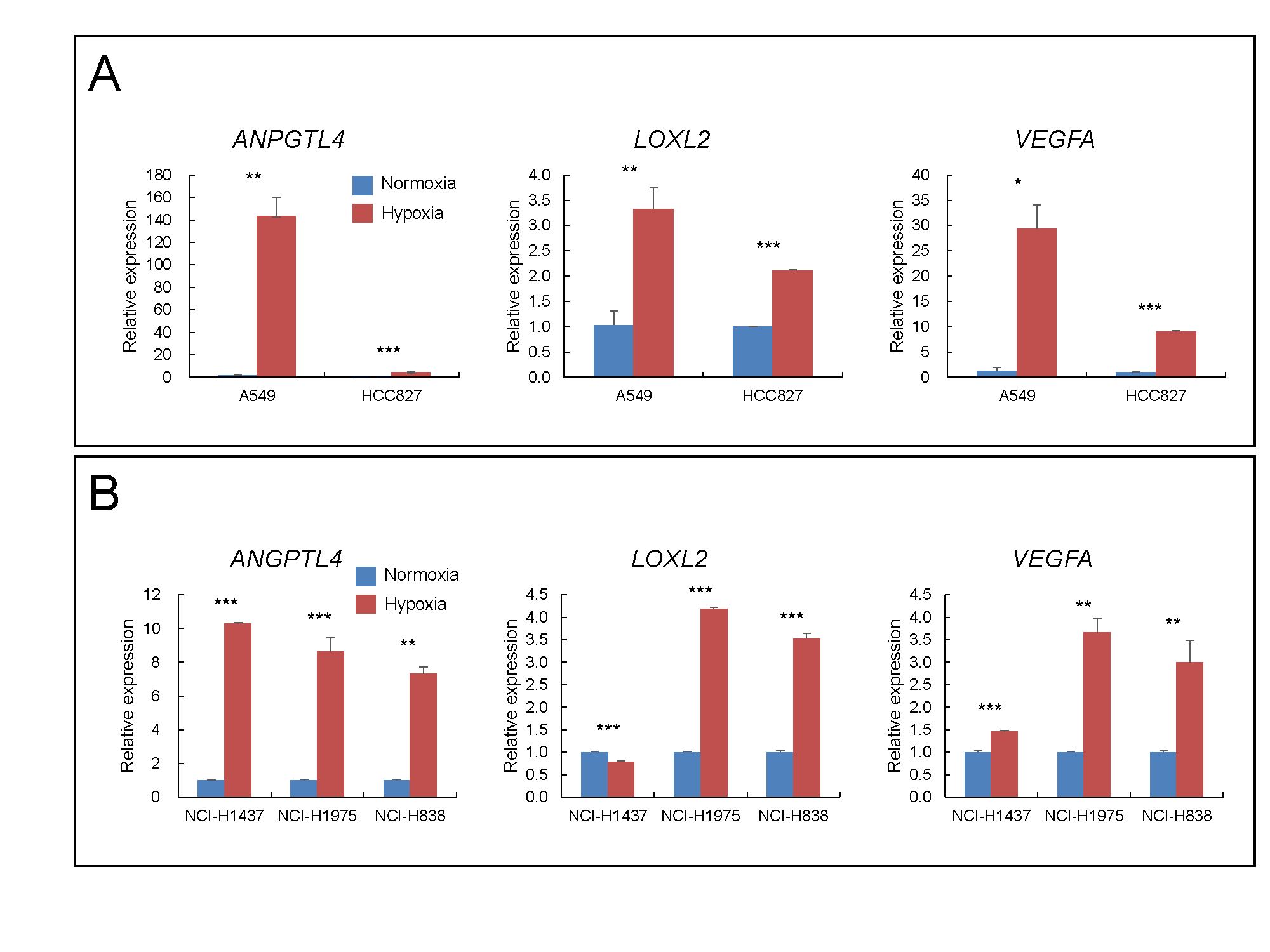


***
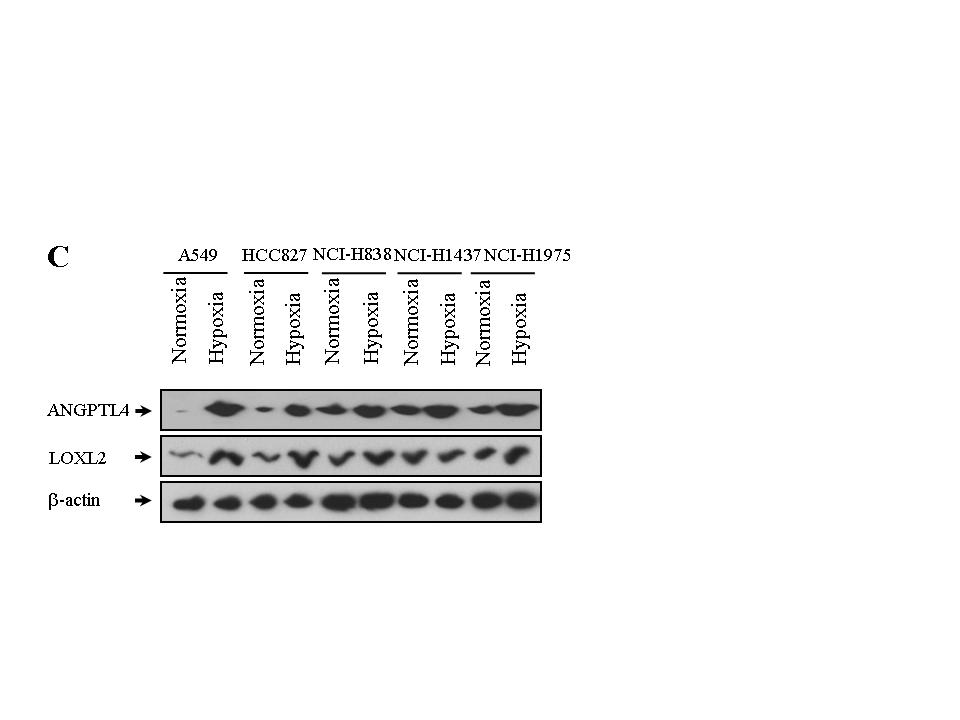
***

**Supplementary Figure 9. The validation of expression changes of three genes (*ANGPTL4*, *LOXL2* and *VEGFA*) in five cell lines.** (**A**) The relative expression of *ANGPTL4, LOXL2* and *VEGFA* after hypoxia induced in A549 and HCC827. (**B**) The mRNA expression of *ANGPTL4, LOXL2* and *VEGFA* genes in another three independent NSCLC cell lines treated with hypoxia, including NCI-H838, NCI-H1437 and NCI-H1975. All the assays were performed in triplicate, and the data are shown as the mean values ± SEM. The asterisks denote significant differences (*, *P*<0.05; ***, *P*<0.001) within experiments, as determined by the Student’s *t*-test. (**C**) The protein expression of ANGPTL4 and LOXL2 were further confirmed by Western blotting in these NSCLC cell lines treated with normoxia and hypoxia.


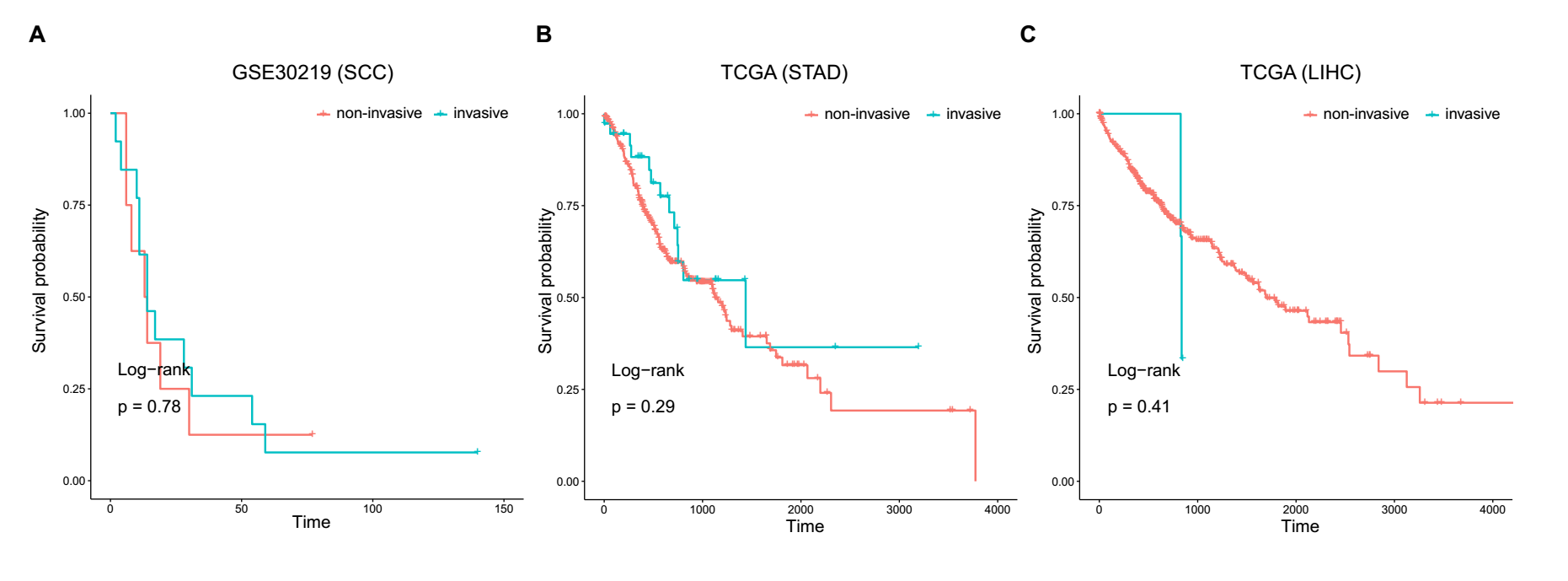


**Supplementary Figure 10.** The survival analyses for four datasets of TCGA, including (A) lung small cell carcinoma (21 samples from GSE30219), (B) stomach adenocarcinoma (TCGA_STAD) and (C) hepatocellular carcinoma (TCGA_LIHC).
